# Giant gastric trichobezoar in a young female with Rapunzel syndrome: case report

**DOI:** 10.11604/pamj.2017.27.252.9110

**Published:** 2017-08-04

**Authors:** Mohamed Hamid, Youssef Chaoui, Mohamed Mountasser, Farid Sabbah, Mohammed Raiss, Abdelmalek Hrora, Mouna Alaoui, Mohammed Ahallat, Safaa Chaouch, Houria Ouazzani

**Affiliations:** 1 Department of Surgery C, Hôpital Ibn-Sina, Rabat, Faculté de Médecine et de Pharmacie Rabat, Mohammed V University Souissi, Rabat, Morocco; 2 Department of Gastroenterology B, Hôpital Ibn-Sina, Rabat, Faculté de Médecine et de Pharmacie Rabat, Mohammed V University Souissi, Rabat, Morocco

**Keywords:** Rapunzel syndrome, giant, trichobezoar, trichotillomania, malnutrition

## Abstract

Rapunzel syndrome is an extremely rare complication of a gastric trichobezoar in. We report here the unusual case of a case of a 16 years old girl presented with severe abdominal pain and vomiting. Clinical examination revealed a malnourished girl, with presence of a mobile and sensitive abdominal mass of 20x15 cm witch filled the upper quadrant. An abdominal computed tomography scan showed a heterogeneous mass occupying the whole stomach cavity with extension into the third portion of the duodenum. A diagnostic of giant trichobezoar is suspected after further questioning reveling a trichotillomania, trichophagia and onychophagia. The removal of the trichobezoar endoscopically failed and it was possible to pull only few fibers of hair to comfort the diagnostic. She was subjected to an exploratory laparotomy. An antral gastrostomy were performed and a 25x10x7 cm trichobezoar was extracted. The patient had an uneventful postoperative outcome and was derived to psychiatry. Rapunzel syndrome is an uncommon trichobezoar, it’s commonly found in young females usually with an underlying psychiatric disorder. Management requires gastrotomy. A psychiatric assessment and a long-term follow-up are advocated as a regular part of treatment to prevent recurrence.

## Introduction

A trichobezoar is an agglomeration of hair which accumulate and remain within the gastrointestinal tract. Trichobezoars often coexist with learning disabilities or psychiatric illness [1], in Rapunzel syndrome, the foreign material extends through the pylorus into the small intestine. In this situation, the trichobezoar may cause significant complications [2–4]. We report here the unusual case of a 16 years old girl presented with giant trichobezoar revealed by severe abdominal pain malnutrition.

## Patient and observation

**Figure 1:**
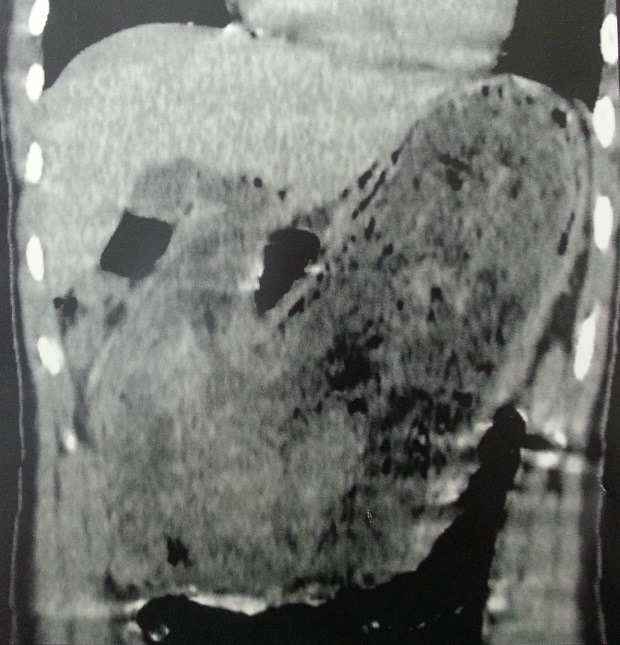
Preoperative CT scan: frontal image of the gastro-duodenal trichobezoar

**Figure 2:**
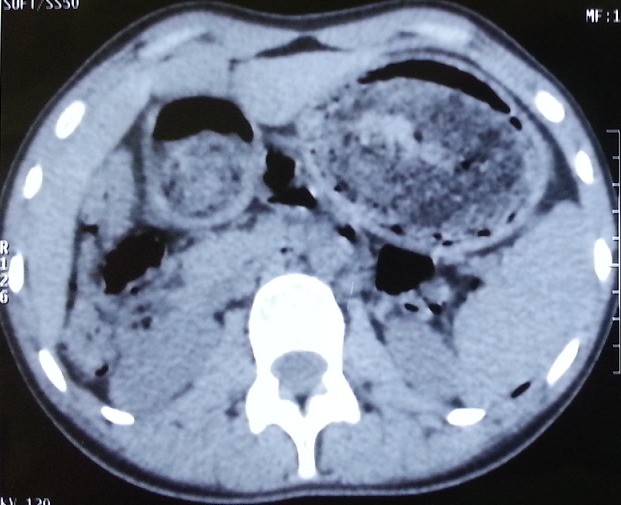
Preoperative CT scan: coronal image of the gastro-duodenal trichobezoar

**Figure 3:**
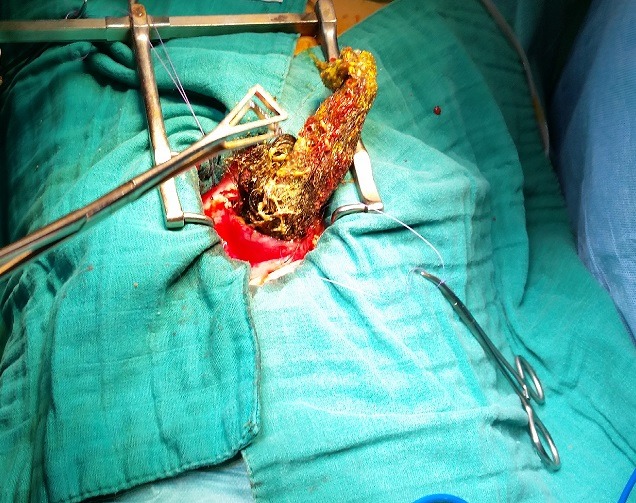
Trichobezoar being delivered

**Figure 4:**
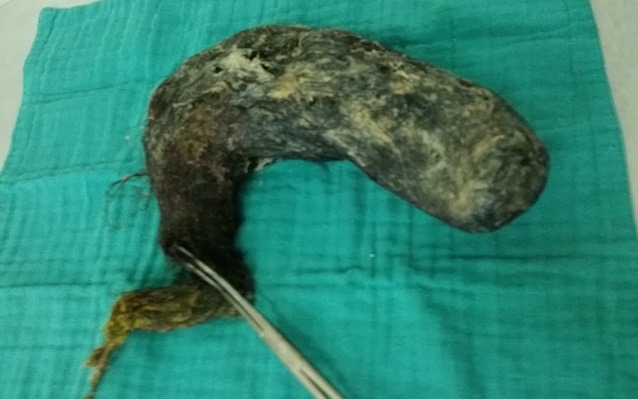
Giant trichobezoar

A 16-year-old female was referred to our surgical clinic with abdominal pain and vomiting of one year duration. Her personal history revealed trichophagia and onychophagia. Clinical examination revealed a malnourished girl, with a mobile well-defined mass occupying the upper half of the abdomen. The mass was not tender and was firm in consistency. There was also evidence of paleness of mucosa and skin. Laboratory test results showed a leukocytosis of 6100/mm, hypochromic microcytic anemia (hemoglobin, 8.2 g/dL), hypoproteinemia (43 g/L) and hypocholesterolemia (1.45 g/L); with normal liver chemistries and lipase. The computed tomography (CT) scan revealed a huge gastric distention with well-circumscribed and heterogeneous mass occupying the whole stomach cavity with extension into the third portion of the duodenum (Figure 1 and Figure 2). Upper gastrointestinal endoscopy pull only few fibers of hair to comfort the diagnostic. The patient underwent surgery, and through upper midline incision, an antral gastrotomy was done. A giant trichobezoar was identified and was removed (Figure 3). There was a long tail of hair extending through the pylorus into the proximal jejunum, the spacemen takes the shape of the stomach and duodenum (Figure 4). By this feature the diagnosis was clear of a Rapunzel syndrome. The gastrotomy was closed with continuous Vicryl 3-0. The patient had an uneventful postoperative course and was discharged after 5 days. The patient was also referred to psychiatric follow-up.

## Discussion

A bezoar is an agglomeration of food or foreign material which accumulate and remain within the gastrointestinal tract. They have been classified into four types: phytobezoars (caused by vegetables), trichobezoars (caused by hair), lactobezoars (caused by milk curds) and miscellaneous (caused by medications, tissue papers, shellac, tar, sand or fungus) [1]. Trichobezoars often coexist with learning disabilities or psychiatric illness [4], it is usually the result of compulsory pulling out (trichotillomania) and eating one’s own hair (trichophagia). The disorder is predominantly observed in adolescents, more frequently in girls than boys [4, 5].

A bezoar is an agglomeration of food or foreign material which accumulate and remain within the gastrointestinal tract. They have been classified into four types: phytobezoars (caused by vegetables), trichobezoars (caused by hair), lactobezoars (caused by milk curds) and miscellaneous (caused by medications, tissue papers, shellac, tar, sand or fungus) [1] Trichobezoars often coexist with learning disabilities or psychiatric illness [5], it is usually the result of compulsory pulling out (trichotillomania) and eating one’s own hair (trichophagia). The disorder is predominantly observed in adolescents, more frequently in girls than boys [5, 6].

Trichobezoars are ineffectively moved by peristalsis due to its smooth surface and poorly digested keratinaceous substance. As a result, the hair becomes matted into a ball and is retained in the folds of upper digestive tract. The ball can reach sizes sufficient to cause stomach distension and chronic malnutrition [7]. In Rapunzel syndrome, the trichobezoar extends through the pylorus into the small intestine. Fewer than a hundred cases of Rapunzel syndrome have been reported in the literature since the initial description in 1968 [6, 8, 9] .

The most common presenting symptoms include abdominal pain, nausea, and vomiting, which occur in 33-37% of patients [7]. In the clinical examination, a well-defined abdominal mass that is smooth, firm and mobile in the epigastric area is found in 85% of patients. Anemia and hypoalbuminemia have also been described [10]. Less commonly, trichobezoars may cause intermittent small bowel obstruction, duodenojejunal fissuration [3] pancreaticobiliary obstruction, peritonitis or pancreatitis [4]. Most of the patients deny any history of trichotillomania or trichophagia, even when specifically asked [6]. The alopecia may also be noted in these patients [11]. In this case, the young patient admits having the habit of eating her hair and it is conscious of the disorder.

A CT scan represented the examination of choice. It can delineate a well-defined oval intraluminal mass with air bubbles retained within the interstice or a homogenous mottled appearance in the region of the stomach or intestine [12] . In this case, the interpretation of the CT in the clinical context is pathognomonic. Endoscopy remains the examination of choice in the diagnosis of intragastric trichobezoar as it allows visualizing the hair threads and trying an extraction.

Various therapeutic modalities have been proposed to treat trichobezoar. Non-surgical extraction by endoscopy or dissolution by Papain syrup often fails and may lead to severe complications [3]. Surgical options have been modified with the advent of laparoscopy, its effectiveness of in combination with a small laparotomy has been reported [13] however, the size of the mass limit this approche in this case. We chose a relatively small antral gastrotomy by a small medline incision that precluded en bloc the evacuation of the trichobezoar. The piecemeal evacuation technique is, as noted by others [14], tedious and the smell is nauseous.

## Conclusion

Rapunzel syndrome is an uncommon trichobezoar, it’s commonly found in young females usually with an underlying psychiatric disorder. Management requires gastrotomy. A psychiatric assessment and a long-term follow-up are advocated as a regular part of treatment to prevent recurrence.

## Competing interests

The authors declare no competing interests.
